# Cyanobacterial biofuels: new insights and strain design strategies revealed by computational modeling

**DOI:** 10.1186/s12934-014-0128-x

**Published:** 2014-09-19

**Authors:** Philipp Erdrich, Henning Knoop, Ralf Steuer, Steffen Klamt

**Affiliations:** Max Planck Institute for Dynamics of Complex Technical Systems, Sandtorstraße 1, Magdeburg, 39106 Germany; Humboldt-University of Berlin, Institute for Theoretical Biology, Invalidenstraße 43, Berlin, 10115 Germany

**Keywords:** Biofuels, Metabolic engineering, Cyanobacteria, Minimal cut sets, Photosynthesis, *Synechocystis* sp. PCC 6803

## Abstract

**Background:**

Cyanobacteria are increasingly recognized as promising cell factories for the production of renewable biofuels and chemical feedstocks from sunlight, CO2, and water. However, most biotechnological applications of these organisms are still characterized by low yields. Increasing the production performance of cyanobacteria remains therefore a crucial step.

**Results:**

In this work we use a stoichiometric network model of *Synechocystis* sp. PCC 6803 in combination with CASOP and minimal cut set analysis to systematically identify and characterize suitable strain design strategies for biofuel synthesis, specifically for ethanol and isobutanol. As a key result, improving upon other works, we demonstrate that higher-order knockout strategies exist in the model that lead to coupling of growth with high-yield biofuel synthesis under phototrophic conditions. Enumerating all potential knockout strategies (cut sets) reveals a unifying principle behind the identified strain designs, namely to reduce the ratio of ATP to NADPH produced by the photosynthetic electron transport chain. Accordingly, suitable knockout strategies seek to block cyclic and other alternate electron flows, such that ATP and NADPH are exclusively synthesized via the linear electron flow whose ATP/NADPH ratio is below that required for biomass synthesis. The products of interest are then utilized by the cell as sinks for reduction equivalents in excess. Importantly, the calculated intervention strategies do not rely on the assumption of optimal growth and they ensure that maintenance metabolism in the absence of light remains feasible. Our analyses furthermore suggest that a moderately increased ATP turnover, realized, for example, by ATP futile cycles or other ATP wasting mechanisms, represents a promising target to achieve increased biofuel yields.

**Conclusion:**

Our study reveals key principles of rational metabolic engineering strategies in cyanobacteria towards biofuel production. The results clearly show that achieving obligatory coupling of growth and product synthesis in photosynthetic bacteria requires fundamentally different intervention strategies compared to heterotrophic organisms.

**Electronic supplementary material:**

The online version of this article (doi:10.1186/s12934-014-0128-x) contains supplementary material, which is available to authorized users.

## Background

Increasing requirements for food, feed and chemical raw materials constitute one of the grand challenges of the 21st century. To overcome the massive problems associated with the use of fossil resources, products derived from cyanobacteria are increasingly recognized as a promising source for renewable biofuels and chemical feedstocks. Cyanobacteria, the ancestors of modern-day chloroplasts, are evolutionary old organisms and are the only known prokaryotes that perform oxygenic photosynthesis. As primary producers, cyanobacteria are able to directly convert atmospheric CO _2_ into hydrocarbons suitable as transport fuels and chemical feedstock. As one of their main advantages, many cyanobacteria are able to grow and proliferate also in harsh and extreme environments, including brackish water and in environments with high salinity. The metabolic versatility of cyanobacteria therefore offers the potential to overcome some of the problems associated with plant-derived first generation biofuels, such as the massive requirement for fresh water and the resulting competition of fuel versus food. Correspondingly, there has been considerable interest in biotechnological applications of cyanobacteria [1-5], ranging from the production of bioactive compounds [6-8], secondary metabolites [9,10] and bioplastics (polyhydroalkanoates) [11-17] to the utilization of cyanobacteria for bioremediation purposes [18-20].

Most applications of cyanobacteria for sustainable production, however, are still characterized by low product yield. While proof-of-concept for cyanobacterial biofuel production has been established for a variety of potential fuels, such as hydrogen [21,22], ethanol [23], and isobutanol [24,25], among others, these approaches as yet mostly rely on simple ad-hoc strategies to improve product yield. In this respect, computational methods for calculating a suitable strain design based on genome-scale metabolic models hold great promise to significantly improve product yield and hence establish cyanobacteria as a universal production chassis. Such computational procedures for suggesting suitable genetic manipulations have been extensively developed for heterotrophic micro-organisms [26-30], often revealing complex and non-intuitive genetic intervention strategies that lead to the overproduction of a desired metabolite [31,32]. Successful intervention strategies usually aim to stoichiometrically couple biomass production to the synthesis of the desired product, thereby making the synthesis of a value-added product an obligatory byproduct of cellular growth. Although several genome-scale stoichiometric metabolic models of cyanobacteria have been published in the last years [33-39], applications of such design principles to phototrophic metabolism have as yet been scarce. In particular, most previous approaches did not succeed to identify suitable coupling strategies for phototrophic growth or were restricted to cyanobacteria grown heterotrophically on an additional carbon source [40,41].

A systematic study clarifying whether growth-coupled production of biofuels with cyanobacteria is feasible or not by suitable interventions and, if so, revealing the key principles behind such strain designs and clarifying major differences to heterotrophic organisms is thus an urgent need. The purpose of this work is therefore to identify and analyze suitable genetic intervention strategies for the overproduction of biofuels, in particular ethanol and isobutanol, based on a metabolic network model of the cyanobacterial strain *Synechocystis* sp. PCC 6803 [33,39]. We employ two different computational methods, CASOP [42] and Constrained Minimal Cut Sets [29], to determine promising intervention strategies. Differing from previous efforts, we focus on phototrophic growth in the absence of organic carbon and explicitly account for the diurnal rhythm of cyanobacteria. That is, we ensure that all identified intervention strategies lead to increased production rates under phototrophic conditions while retaining a functional metabolism to meet cellular demands for maintenance processes at night.

Our results demonstrate that intervention strategies exist that enforce coupled biomass and product synthesis with high yields of ethanol and isobutanol in *Synechocystis* sp PCC 6803. We enumerate all knockout strategies in a reduced version of the genome-scale network model and apply recently developed methods [43] to identify a large number of the smallest suitable intervention strategies also in the full genome-scale model. Our computational analysis reveals that relevant strategies require a relatively large number of interventions which we analyze in more detail with respect to their physiological consequences. We show that most promising strategies exploit differences in the ATP/NADPH-requirements for synthesizing biomass and biofuels, respectively. Accordingly, many intervention strategies target routes in the electron transport chain to redistribute fluxes in photosynthetic linear and alternate electron flow pathways. Furthermore, we also show that a moderately increased ATP turnover induced, for example, by ATP futile cycling or other ATP wasting mechanisms is a promising target to achieve increased product yields.

## Methods

### Metabolic network models

All computations made herein are based on the genome-scale network model (GN) of *Synechocystis* sp. PCC 6803 of Knoop *et al*. [39]. We took the original genome-scale model (abbreviated by GN) and added the ferredoxin plastoquinone reductase (FQR) reaction as well as the Mehler and related reactions [44,45] as putative additional alternate electron transport pathways. To allow for the synthesis of ethanol and isobutanol, the pyruvate-decarboxylase [23] and the reactions of the isobutanol synthesis pathway, as well as excretion reactions for both metabolites, were incorporated into the model. To account for night metabolism, a reaction to mobilize stored intracellular glycogen was added. Reactions from isozymes are considered only once in the model. Furthermore, we consider water and cytoplasmic protons as external metabolites, whereas protons in the thylakoid and periplasmic space establishing the proton-motive force needed for ATP formation via ATP synthase are explicitly balanced. In total, the GN network contains 600 reactions and 518 (internal) metabolites.

To allow exhaustive elementary modes analysis, we derived also a reduced network (abbreviated RN) from the GN model comprising 505 reactions and 487 (internal) metabolites. The RN version is a true subnetwork of GN where some futile cycles and alternate electron pathways with presumably lower importance were removed. In contrast to the GN model, all electron transport processes in the RN model take place in a single membrane compartment to avoid duplicate reactions in the cytoplasmic and in the thylakoid membrane. Both the GN and RN model contain all main metabolic pathways, including photosynthetic (linear, cyclic, pseudocyclic) electron flows, respiratory pathways (via the cytochrome *bd* quinol oxidase (Cyd) and the *aa*3-type cytochrome *c* oxidase (Cox)), the Calvin-Benson-Bassham (CBB) cycle, glycolysis, pentose-phophate pathway, photorespiration, amino acid synthesis, lipid and fatty acid metabolism, biosynthesis of peptidoglycan, chlorophylls, carotenoids, terpenoids, quinones and tocopheroles, thiaminediphosphates, as well as the synthesis of several co-factors, vitamins and several stress related-pathways [39]. A pseudo-reaction with cumulative stoichiometries for biomass synthesis allows simulation of growth in both models. Selected reactions contained in RN and GN model discussed later in conjunction with the computational results are referenced in Table 1 (the corresponding reaction IDs used in the model of Knoop et al. [39] can be found in Additional file 1: Table S1).

**Table 1:** **List of selected reactions contained in the RN and GN model and associated reaction IDs that will be used when discussing results of CASOP and cut set analysis**

**Reaction ID**	**Reaction**
**(enzyme/name)**	
R1 (NDH1)	NADPH + 5 H ^+^ + PQ ⇒ NADP ^+^ + PQH _2_ + 4 H$^{+}_{\text {tll}}$
R2 (NDH1)	NADPH + 4 H ^+^ + PQ + H _2_O + CO _2_ ⇒ NADP ^+^ + PQH _2_ + 4 H$^{+}_{\text {tll}}$ + HCO3 ^−^
R3 (Cox)	4 Reduced plastocyanin + O _2_ + 8 H ^+^ ⇒ 4Oxidized plastocyanin + 4 H$^{+}_{\text {tll}}$ + 2 H _2_O
R4 (Cyd)	2 PQH _2_ + O _2_ + 4 H ^+^ ⇒ 2 PQ + 4 H$^{+}_{\text {tll}}$ + 2 H _2_O
R5 (Mehler)	O _2_ + Reduced ferredoxin ⇒ Oxidized ferredoxin + O$_{2}^{-}$
R6	2 O$_{2}^{-}$ + 2 H ^+^ ⇒ H _2_ *O*_2_ + O _2_
R7 (FQR)	PQ + 2 Reduced ferredoxin + 2 H ^+^ ⇒ 2 Oxidized ferredoxin + PQH _2_
R8	D-Ribulose 1,5-bisphosphate + O _2_ ⇒ 3-Phospho-D-glycerate + 2-Phosphoglycolate
R9	O _2_⇒ O _2cax_
R10	2-Phosphoglycolate + H _2_O ⇒ Glycolate + Orthophosphate
R11	2-Phosphoglycolate _cax_⇒ 2-Phosphoglycolate
R12	NADPH + NAD ^+^ ⇔ NADP ^+^ + NADH
R13	ATP + Acetate ⇔ ADP + Acetyl-phosphate
R14	sn-Glycerol 3-phosphate + NAD ^+^ ⇔Glycerone-phosphate + NADH + H ^+^
R15	ATP + Glycerol ⇒ ADP + sn-Glycerol3-phosphate
R16	Glycerol + NADP ^+^ ⇔ D-Glyceraldehyde + NADPH + H ^+^
R17	D-Glyceraldehyde + NAD ^+^ + H _2_O ⇔D-Glycerate + NADH + H ^+^
R18	5,10-Methylenetetrahydrofolate + Glycine + H _2_O ⇔ Tetrahydrofolate + L-Serine
R19	Glycine + Tetrahydrofolate + NAD ^+^ ⇒5,10-Methylenetetrahydrofolate + NH3 + CO2 + NADH + H ^+^
R20	D-Glycerate + NADP ^+^ ⇔ Hydroxypyruvate + NADPH + H ^+^
R21	L-Serine + Glyoxylate ⇔ Hydroxypyruvate + Glycine
R22	Glycine + 2-Oxoglutarate ⇔ Glyoxylate +L-Glutamate
R23	3-Phospho-D-glycerate + NAD ^+^ ⇔3-Phosphonooxypyruvate + NADH + H ^+^
R24	O-Phospho-L-serine + 2-Oxoglutarate ⇔3-Phosphonooxypyruvate + L-Glutamate
R25	O-Phospho-L-serine + H _2_O ⇒ L-Serine +Orthophosphate
R26	(S)-Malate + NAD ^+^ ⇔ Oxaloacetate + NADH + H ^+^
R27	D-Fructose-6-phosphate + Orthophosphate ⇒ Acetyl-phosphate + D-Erythrose-4-phosphate + H _2_O
R28	D-Xylulose-5-phosphate + Orthophosphate ⇒ Acetyl-phosphate + D-Glyceraldehyde-3-phosphate + H _2_O
R29	Ethanol ⇒ Ethanol _ex_
R30	Acetaldehyde + NADPH + H ^+^⇒ Ethanol + NADP ^+^
R31	Pyruvate ⇒ CO _2_ + Acetaldehyde
R32	ADP + Phosphoenolpyruvate ⇒ ATP + Pyruvate
R33	CO _2_ ⇒ CO _2*ext*_
R34	ATP + D-Fructose-6-phosphate ⇒ ADP + D-Fructose-1,6-bisphosphate
R35 (ATPm)	1 ATP + H _2_O ⇒ 1 ADP + Orthophosphate
R36	Cyanophycine + 2 H _2_O ⇒ 1 L-Aspartate + 1 L-Arginine + Cyanophycin-polymer
R37	1 L-Aspartate + 1 L-Arginine + 2 ATP + Cyanophycin-polymer ⇒ Cyanophycin + 2 Orthophosphate + 2 ADP
R38	ATP + Sedoheptulose-7-phosphate ⇒ ADP + Sedoheptulose-1,7-bisphosphate

**Figure 1:**
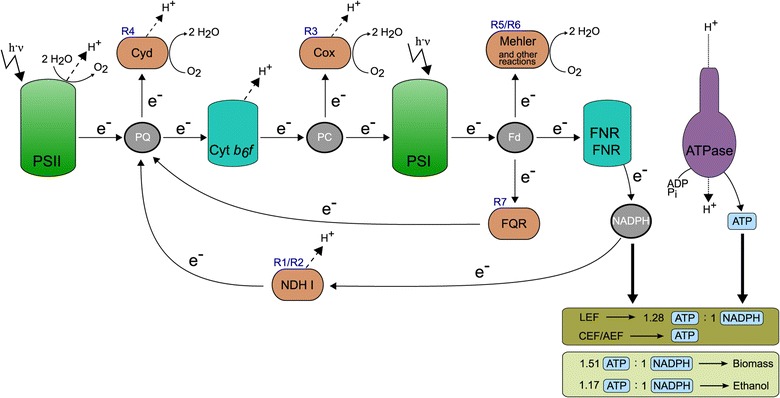
**Selected key reactions of linear and alternate electron flow pathways in** ***Synechocystis*** **sp.** PCC 6803 contained in the RN and GN model. Dashed arrows with *H* ^+^ represent release or pumping of protons into the thylakoid lumen. The resulting proton motive force is then used for ATP synthesis via ATPase. The two boxes at the bottom (right) display ATP and NADPH stoichiometries of electron flow pathways and for biomass and ethanol synthesis. Abbreviations: PSI/PSII: photosystem I and II, Cyt *b*_6_ *f*: cytochrome *b*_6_ *f*, Cox: cytochrome *c* oxidase, Cyd: cytochrome *bd*-type quinol oxidase, FNR: ferredoxin NADP reductase, ATPase: ATP synthase, FQR: ferredoxin quinone reductase, NDH I: NADPH dehydrogenase I, PQ: plastoquinone, PC: Plastocyanin, Fd: Ferredoxin, LEF: linear electron flow, CEF: cyclic electron flow, AEF: alternate electron flow, e ^−^: electrons.

Some specific reaction (or enzyme) names used in this study (e.g., in Figure 1) are also given. The corresponding reaction IDs used in the model of Knoop et al. [39] can be found in Additional file 1: Table S1.

The RN model version can be seen as a core model of the GN version enabling us to compute the full set of elementary modes required for the CASOP procedure and for enumerating the complete set of intervention strategies (minimal cuts sets, see below). Since much of the computational results will center around electron flows in the membrane, Figure 1 summarizes the key reactions of the electron transport chain. Whereas the RN model will be used for exhaustive analysis of elementary modes and minimal cut sets, the full GN version still allows us to enumerate and analyze more than 1000 of the smallest intervention strategies.

An SBML representation of both models, GN and RN, are contained in the Additional files 2 and 3. All calculations discussed herein were performed with the MATLAB-toolbox *CellNetAnalyzer* [46].

### Elementary modes and (constrained) minimal cut sets

Elementary Modes (EMs) provide a suitable concept for a functional characterization of metabolic reaction networks by means of minimal functional units [47,48]. Formally, EMs are non-decomposable steady state flux vectors meaning that no subset of the reactions involved in an EM can generate a feasible steady state flux distribution. EMs are useful to study functional properties of metabolic networks [48]. As a key property, conical (non-negative linear) combinations of EMs generate the entire space of steady-state flux distributions. Furthermore, EMs enable one to identify all optimal and suboptimal pathways that allow for the synthesis of biomass or a product of interest. Several methods for calculating metabolic engineering strategies rely on EMs, among them minimal cut sets and CASOP used herein.

Minimal cut sets (MCSs) provide a framework for enumerating metabolic intervention strategies disabling certain functions in a metabolic (or stoichiometric) network. Formally, MCSs are (support-)minimal sets of reaction (or gene) knockouts blocking a predefined behavior [49,50]. The easiest way to calculate MCSs is to select a set of *target EMs* representing the functionality to be blocked and to compute subsequently the so-called minimal hitting sets which are sets of reactions that hit each target EM in at least one reaction, thereby disabling all target EMs.

**Figure 2:**
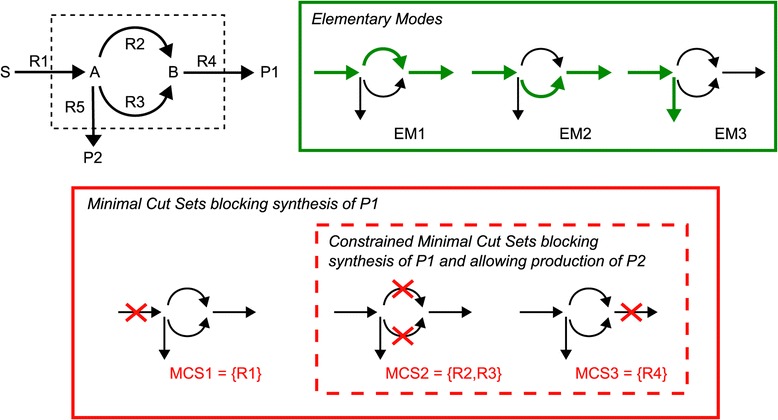
**A simple reaction network with its elementary modes, minimal cut sets and constrained minimal cut sets.** There are three minimal cut sets blocking synthesis of P1 of which two remain as constrained minimal cut sets if production of P2 is a desired function to be kept.

An example is shown in Figure 2. The toy reaction network contains three EMs, two for synthesis of product P1 (EM1, EM2) and one for P2 (EM3) from substrate S. Suppose that synthesis of product P1 in Figure 2 is to be disabled. Accordingly, EM1 and EM2 would be selected as target modes and the resulting three MCSs are {R1},{R2,R3} and {R4}. Each MCS is minimal as no subset of it would ensure blocking of P1 synthesis.

The approach of *constrained* MCSs (cMCSs) goes one step further [29]. Since MCSs may block undesired but also desired metabolic behaviors, the approach of cMCSs allows the specification of both *target* and *desired EMs*. An cMCS hits all target EMs and keeps at least one of the desired EMs feasible. Coming back to the example in Figure 2, consider a situation in which production of P1 is to be repressed while synthesis of P2 is demanded to be possible. As in the previous example we select EM1 and EM2 as target modes, but in addition we set EM3 as (the only) desired mode. The cMCSs to this problem are {R2,R3} and {R4}. {R1} is an MCS but not a cMCS because it hits the only desired mode. It generally holds true, that the cMCSs are a subset of the MCSs, the latter being computed with an empty set of desired EMs.

As mentioned above, MCSs and cMCSs for a given intervention problem can conveniently be computed from the corresponding sets of target EMs and desired EMs. However, in large networks, the determination of EMs is often not possible. To compute cMCSs also in the genome-scale model GN, we employ a recently developed algorithm [43] by which at least the smallest cMCSs can be directly computed from the network without the requirement to compute in an initial step the complete set of EMs. This novel approach relies on the duality between EMs and MCSs [51]. Desired and undesired functionalities are described by appropriate linear equalities and inequalities. The procedure delivers iteratively the smallest, the second smallest, …, the k-th smallest MCS, where smallest is understood in terms of the number of required knockouts. In this way a large number of the smallest MCSs can be determined also in large networks. In order to keep only the subset of valid *constrained* MCSs, each found MCS is tested by a simple optimization problem whether its application does not disable the desired functions [43].

### Enforcing coupled product and biomass synthesis and consideration of day/night cycles

As described above we are interested in strategies that couple under photoautotrophic conditions biomass synthesis with the obligate synthesis of biofuels (here: ethanol or isobutanol). In addition, these strategies must not disable the cell to perform maintenance metabolism in the absence of light. To ensure the latter, we demand that the cMCSs must allow regeneration of ATP from the storage compound glycogen in the absence of light. For the analysis of the reduced model RN (where all EMs are calculable), we therefore proceed as follows: We compute the EMs separately for day (photoautotrophic regime) and for night (dark metabolism dominated by ATP regeneration based on intracellular glycogen). Two sets of desired EMs are then selected, one set for the photoautotrophic regime and one for dark metabolism. The desired day EMs are evaluated according to two thresholds, the first specifying the requested minimum biomass yield and the second the minimum requested product yield. The set of desired day EMs comprises all EMs where the biomass and the product yield are above the respective thresholds. The desired night EMs comprise all EMs that allow net production of ATP under night conditions. We note that the set of night EMs also includes the possibility of product synthesis, although this is not enforced by the algorithm. Regarding the undesired behaviors, the *target* EMs to be blocked by the cMCSs are given by all photoautotrophic EMs with a product yield below the defined threshold.

With this partitioning, the resulting cMCSs will hit (disable) all target modes and keep at least one EM of each of the two sets of desired (day and night) EMs feasible. When computing the smallest cMCSs in the GN, we calculate first the smallest MCSs blocking the undesired behaviors (via the method described in [43]) and, in a second step, identify the valid subset of *constrained* MCSs which keep the desired day and night functionalities feasible.

### CASOP

CASOP (Computational Approach for Strain Optimization aiming at high Productivity) is another EM-based framework for target identification [42]. In contrast to the cut set approach, CASOP identifies single knockout *and* overexpression candidates, and it seeks to find a balance between high-yield and high-productivity strategies. Briefly, EMs are computed for the wild-type behavior (usually characterized by growth) as well as for the desired phenotype (most substrate is converted to the product of interest while some biomass is also synthesized). These two sets of EMs are then compared resulting in a score between −1 and 1 for each reaction. The largest positive scores indicate overexpression candidates whereas negative values expose potential knockout candidates.

## Results and Discussion

### The balance of ATP and NADPH as a key target for product synthesis

The importance of the balance between ATP and NADPH synthesis in photosynthetic organisms for achieving metabolic homeostasis is well-known and its relevance for increased production of certain chemicals has also been discussed [52]. The linear electron flow (LEF) through the photosystem II (PSII) and photosystem I (PSI) in cyanobacteria generates an ATP/NADPH ratio of approximately 1.28 (9 ATP and 7 NADPH) whereas binding one molecule of CO _2_ in the CBB cycle consumes 3 ATP and 2 NADPH yielding thus a ratio of 1.5. This imbalance can be resolved by the cell by a number of alternate electron flow (AEF) pathways [36,53] which recycle electrons leading to a net production of ATP without generation of NADPH (Figure 1). These AEF pathways include cyclic photosynthesis via NAD(P)H-dehydrogenases and a FQR reaction as well as the pseudocyclic water-water-cycles via the Mehler and the Mehler-like [54] reaction. Furthermore, respiration under photosynthetic conditions may further modulate the overall ATP/NADPH ratio of the electron transport chain. It is straightforward to compute the ratio of ATP/NADPH net requirements if the cell would only produce a certain biofuel; for the case of ethanol it amounts to 1.17 (7 ATP, 6 NADPH) [55]. To the best of our knowledge, the ATP/NADPH ratio required for the production of biomass has not been determined so far. Calculating such a ratio is not trivial, since many ATP-requiring processes are difficult to quantify and their magnitude strongly depends on environmental conditions. To obtain at least a lower bound, ATP and NADPH were provided to the network as external sources. Subsequently we maximized biomass production in the RN model with photosynthesis and respiration disabled. Our calculations show that then an ATP/NADPH ratio of at least 1.51 is required to generate biomass. This value is most likely larger due to various ATP-consuming processes that might not be included in the model. Hence, synthesizing biomass requires a larger ATP/NADPH ratio than the CBB cycle. This finding has important consequences: the gap between the different ratios (1.17, 1.28, 1.5, 1.51) provides a key target for suitable intervention strategies redirecting carbon flow from biomass to ethanol. Accordingly, as detailed below, most of the interventions found by our computational analysis seek to reduce the produced ATP/NADPH ratio thereby favoring product synthesis. In principle, the manipulation of the ATP/NADPH ratio towards smaller values can be achieved by one of the following four mechanisms: (i) lowering ATP production, (ii) increasing ATP consumption, (iii) increasing NADPH production, and (iv) decreasing NADPH consumption. As the ratio of produced ATP/NADPH is largely controlled by the AEFs, we can expect that the latter will become key targets in engineering strategies. Furthermore, since cyanobacteria possess multiple AEFs, a relatively large number of knockouts might be required to block all pathways that can potentially increase the ATP/NADPH production ratio.

### Elementary modes for ethanol production

**Figure 3:**
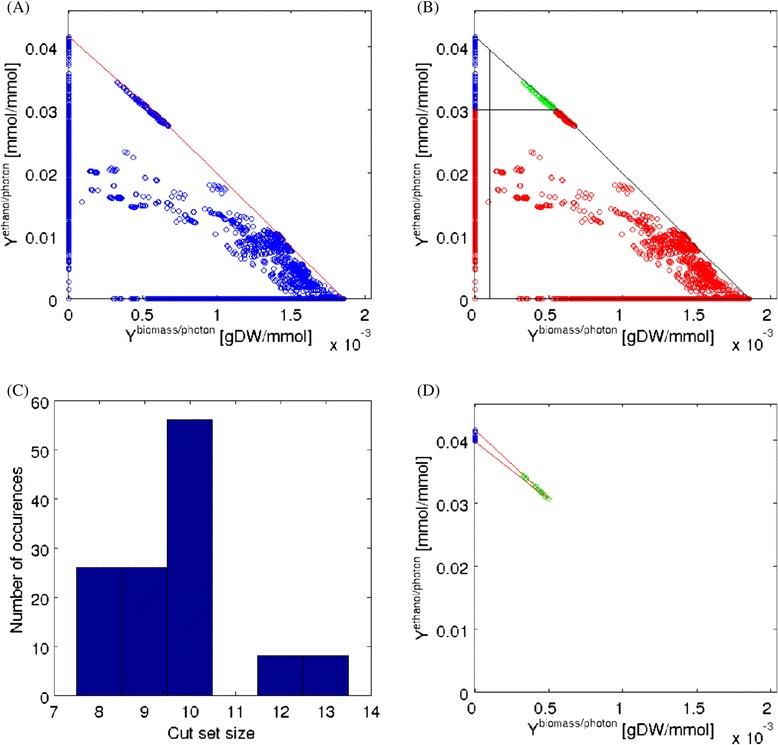
**Elementary modes and constrained minimal cut sets in the RN model.** **(A)** Phenotypic phase plane depicting the specific biomass and ethanol yields of the EMs (day conditions). Each blue circle corresponds to one or several EMs. **(B)** For cMCSs calculation, EMs from (A) are classified as target and desired EMs by specifying thresholds for minimum desired biomass and product yield. Red circles represent target modes (*Y*_ethanol/photon_≤0.03) and green circles desired modes (*Y*_ethanol/photon_>0.03 and *Y*_biomass/photon_≥0.0001). Blue circles indicate modes that are neither target nor desired modes. **(C)** Distribution of the cardinalities of cMCSs calculated from the intervention problem posed in (B) (see also Figure 5). **(D)** Phenotypic phase plane with the remaining EMs of the mutant resulting from a knockout of all reactions contained in cMCS-1 in Figure 5.

We first computed the EMs in the RN model for photosynthetic growth and ethanol production and found that 100803 such EMs exist. Figure 3A shows a phenotypic phase plane projecting for each EM the respective biomass (gDW/mmol photons) and ethanol (mmol ethanol/mmol photons) yield. The convex hull of all these EMs (indicated by the red line) describes the entire space of feasible ratios of biomass and product yields. Circles on the x-axis represent EMs producing exclusively biomass and the maximum biomass yield under optimal conditions is 1.85·10^−3^ gDW/mmol photons. The maximum ethanol yield (upper left corner on the y-axis) is 0.04167 mmol/mmol photons reflecting the theoretical demand of 24 photons per molecule ethanol. With an estimated maximal photon uptake rate of 100 mmol photons/gDW/h [40], the maximum growth and ethanol production rates would be 0.185 h ^−1^ and 4.167 mmol/gDW/h, respectively. As expected, ethanol production reduces the optimal growth yield leading to the characteristic triangle form of the convex hull.

Another important observation that can be made by Figure 3A is the fact, that obligate coupling of product and biomass synthesis can be achieved: coupling is possible if at least one EM exists that has a non-zero yield for both biomass and ethanol and, indeed, many EMs of this kind exist. In the extreme case, one could knockout all reactions not contained in such an EM and the only possible behavior that would remain is the EM itself forcing the coupled behavior. Computing cMCSs, however, will provide a far more elegant and efficient approach to achieve this goal. Figure 3A shows that the theoretical maximum *guaranteed* yield of ethanol under growth-coupled conditions is 0.0344 mmol/mmol photons; this value is obtained from the EM with largest product yield among all EMs with a non-zero biomass yield.

As described in the Methods section, we also determined the EMs for the night scenario (metabolization of stored glycogen) yielding more than 17 million EMs. Hence, compared to photoautotrophic conditions, utilization of a stored carbon source results in a massively increased metabolic diversity, as implied by the larger number of EMs.

### CASOP analysis for ethanol production

To obtain an overview of relevant intervention targets for shifting the cell from wildtype growth behavior to coupled ethanol and biomass synthesis, we applied the CASOP procedure to the EMs under photoautotrophic conditions. As described in the Methods section, CASOP delivers for each reaction a score between −1 and 1 where negative values indicate potential knockout candidates and positive values mark overexpression candidates.

Table 2 shows the top knockout and overexpression candidates for ethanol synthesis together with their CASOP scores. The knockout candidates clearly support our considerations made in a previous section: the proposed reaction deletions mainly target AEF pathways (see Figure 1): (i) respiratory reactions (R3 (Cox), R4 (Cyd); for reaction IDs see Table 1), (ii) reactions involved in cyclic photosynthesis, e.g., of the NADPH dehydrogenase NDH1 (R1, R2) and of FQR (R7), and (iii) reactions of the water-water cycle (Mehler reaction R5, R6). All these reactions may produce ATP without generation of NADPH, some (respiration, water-water cycle) re-reduce oxygen leading in the net to a loss of electrons increasing the ATP/NADPH ratio even further.

**Table 2:** **Top knockout and overexpression (or flux enhancement) candidates for ethanol production as determined by CASOP**

**Top knockout candidates**		**Top overexpression candidates**	
**Reaction ID**	**CASOP score**	**Reaction ID**	**CASOP score**
R1	-0.189	R29	1.000
R3	-0.189	R30	1.000
R7	-0.142	R31	1.000
R4	-0.142	R32	0.298
R2	-0.121	R33	0.173
R5	-0.118	R34	0.128
R6	-0.118	R35	0.096
		R36	0.096
		R37	0.096
		R38	0.096

For reaction IDs see Table 1.

**Figure 4:**
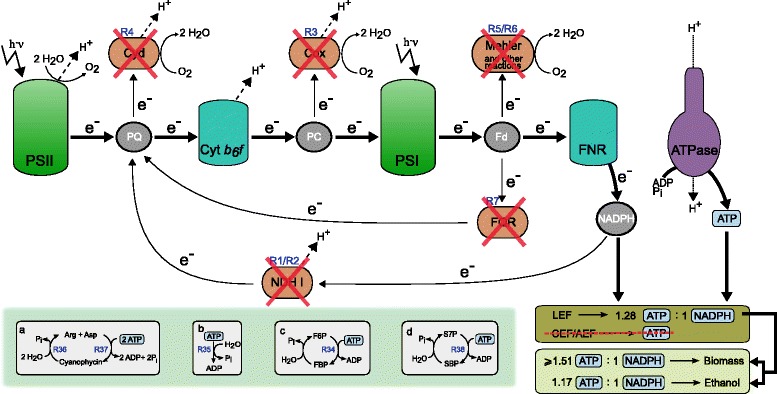
**Intervention strategies suggested by CASOP and cut set analysis in the RN model.** The red crosses represent suggested knockout targets. Cut set analysis reveals that deletion of all these targets blocks all CEF/AEF pathways and leads thus to a fixed ATP/NADPH ratio of 1.28 generated by photosynthesis via the remaining LEF (thick arrows). Ethanol synthesis becomes then mandatory to readjust the ATP/NADPH balance for biomass synthesis. The mechanisms (a)-(d) show overexpression (or flux enhancement) targets suggested by CASOP all of which will enforce an increased turnover (wasting) of ATP. For abbreviations and general explanations see Figure 1.

Regarding the overexpression candidates, a trivial result is that CASOP suggests to enhance the activity of the pyruvate decarboxylase, alcohol dehydrogenase and ethanol excretion reaction (R29, R30, R31) as they constitute the path to ethanol production. Less intuitively, CASOP also proposes to enhance the unspecific ATP maintenance demand reaction (R35) thus suggesting to enhance ATP turnover or to waste ATP. The same strategy lies behind enhancing a cycle around cyanophycin (R36, R37), and behind the phosphofructo-1-kinase (R34) and sedoheptulose-1,7-bisphosphate (R38) reactions all of which force futile cycling and thus loss of ATP (Figure 4). These suggested intervention strategies can be seen as a complementary approach to the top knockout strategies: wasting ATP will lower the ATP/NADPH ratio in the cell which is predicted to have a beneficial effect for ethanol synthesis. CASOP also reveals that an increased CO _2_ export/diffusion is expected to increase ethanol synthesis. In fact, this would again force an energy-consuming pathway by which bicarbonate (HCO$_{3}^{-}$) is taken up and then converted to CO _2_.

### Constrained minimal cut sets for high-yield growth-coupled ethanol production

**Figure 5:**
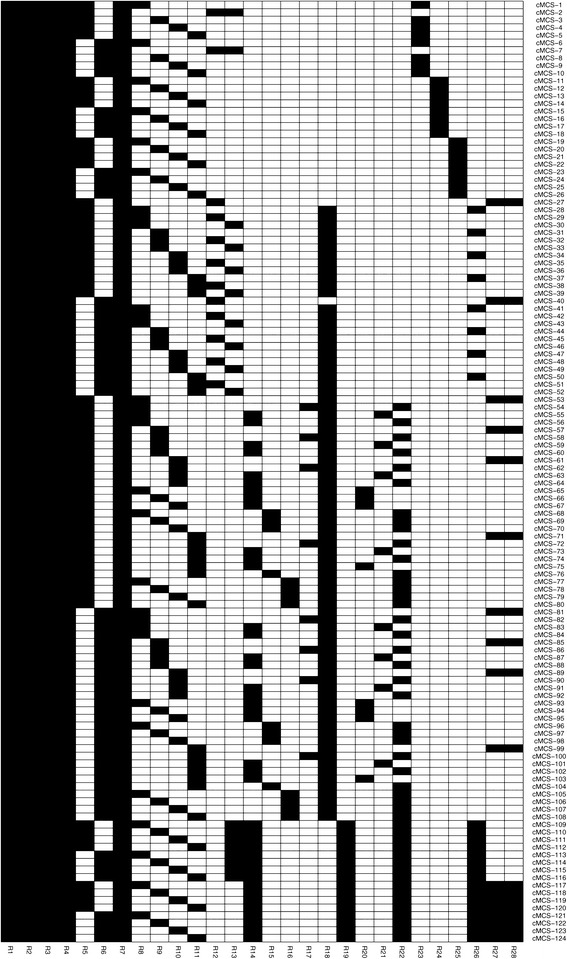
**The complete set of intervention strategies (cMCSs) in the RN model enforcing high-yield growth-coupled ethanol synthesis.** For reaction IDs see Table 1.

We now address the computation of cMCSs (first in the RN and then in the GN model) that will delete all low-ethanol-yield EMs and keep at least some desired EMs exhibiting high product and at least a minimal biomass yield. The threshold for the minimum desired yield is set to *Y*_ethanol/photon_=0.03 for ethanol and *Y*_biomass/photon_=0.0001 for biomass. Hence, all EMs with *Y*_ethanol/photon_≤0.03 are target modes whereas the desired modes fulfill *Y*_ethanol/photon_>0.03 and *Y*_biomass/photon_≥0.0001 (Figure 3B). In addition we demand that the cMCSs must allow ATP synthesis from glycogen under night conditions. We fully enumerated the corresponding cut sets and found in total 124 cMCSs (Figure 5) each comprising 8-13 reaction knockouts (Figure 3C).

As an example, we pick the first cMCSs in Figure 5 (cMCS-1) consisting of 8 cuts. Apparently, many of the suggested knockouts in this cMCS disable reactions which are part of AEF pathways including those that give rise to cyclic (R1, R2, R7) and pseudo-cyclic electron flows (R5) or are involved in respiratory pathways (R3, R4). cMCS-1 also demands to block photorespiration (R8) and reaction R23. Disabling the flux through the 8 reactions contained in cMCS-1 leads to the desired behavior in the model: in order to supply ATP and NADPH for biomass synthesis the cell has no other choice than using the LEF pathway for producing both, because AEF pathways for NADPH-independent ATP-production were disabled. Accordingly, the maximum ATP/NADPH ratio that can be generated by the electron transport chain is now 1.28 and thus below the 1.51 required for biomass synthesis. This leads to a surplus of NADPH which the cell can only oxidize by producing ethanol. Figure 3D shows the remaining (day) EMs (in comparison to the full set of EM in Figure 3B) after deleting the reactions of cMCS-1 confirming again that cell growth is only possible with a simultaneous high-yield synthesis of ethanol. The minimum yield of ethanol would be above 0.03 mmol/mmol photons as requested; this holds for any growth rate/yield the cell would choose from the remaining space of metabolic behaviors.

Analyzing the complete set of 124 cMCSs in Figure 5 reveals that a total of 28 different reactions are used as targets. Five reactions (R1-R4 and R7) are contained in all cMCSs and represent therefore essential cuts to obtain coupled ethanol and biomass synthesis (note that R1 and R2 are catalyzed by the same enzyme NDH1). Deletion of these reactions completely disables respiration and cyclic photosynthesis. Disabling the water-water cycle via the Mehler reaction is also essential but can be realized by removing either R5 or R6. In fact, we found that deleting these mechanisms/enzymes blocks all major CEF/AEF pathways (Figure 4) and is therefore already sufficient to obtain growth-coupled ethanol production with a minimum ethanol yield of 0.027 mmol/mmol photons. Reaching our predefined minimum ethanol yield of 0.03 mmol/mmol photons requires disruption of some further pathways that may unfavorably modulate the ATP and NAD(P)H stoichiometries. This includes, in particular, pathways involved in photorespiration but also others such as the phosphoketolase reactions R27 and R28.

Generally, the cMCSs confirm the knockout targets proposed by CASOP: all reaction knockout candidates in Table 2 are essential cuts in Figure 5, with the exception that either R5 or R6 must be deleted.

Regarding the night behavior, we found that all cMCSs enforcing the desired day behavior still allow synthesis of ATP from intracellular glycogen. Since respiratory pathways are disabled by all cMCSs, the cell can synthesize ATP only by a fermentative pathway. As a beneficial side effect, ethanol becomes thereby a mandatory fermentation product further increasing its overall yield. As it is known that many cyanobacteria engage in fermentative metabolism in the absence of light and oxygen, such a strategy can indeed be expected to provide sufficient energy for maintenance requirements [56].

#### Constrained MCSs in the full genome-scale network model

In addition to full enumeration of cMCSs in the reduced network model RN, we also enumerated the smallest cMCSs in the genome-scale model GN using the same constraints for the desired minimum yields for ethanol and biomass and with the requirement that the cMCSs must allow ATP synthesis from glycogen under night conditions.

We found that the minimum number of required cuts is 14 and we were able to identify all cMCS of size 14 (66), 15 (244) and 16 (1254) yielding in total 1564 cMCSs. We first noticed that all computed cMCSs have seven (essential) reaction knockouts in common: R1, R3, R4, R7, which were already essential in the RN model, and, additionally, the reaction of the NDH2 (NADH-dependent dehydrogenase), the Mehler-like reaction and the reaction ‘Proline + PQ ⇒ 1-Pyrroline-5-carboxylate + PQH2’. These three reactions were not contained in the RN model and give rise to additional AEF pathways and must therefore be blocked to obtain coupled ethanol and biomass synthesis. We also found that reaction R2, which was an essential cut in the RN model, is not necessarily to be removed in the GN model. The reason is that some desired behaviors in the GN model exist that involve this reaction implying that a removal of this reaction is not essential in all strain designs.

Apart from the essential cuts mentioned above, several additional pathways need to be blocked in the GN model because they can potentially increase the ATP/NADPH balance in the cell. Non-essential cuts are replaceable in other cMCSs, for instance, if an AEF pathway involves a cycle with several reactions then one of these reactions need to be blocked. One such example comprises the transhydrogenase reaction together with the two reactions ‘sn-Glycerol 3-phosphate + PQ ⇒ Glycerone phosphate + PQH2’ and ‘sn-Glycerol 3-phosphate + NAD ^+^ ⇔ Glycerone phosphate + NADH + H ^+^’. These reactions establish another AEF pathway via which electrons from NADPH cycle back to the electron transport chain thereby pumping protons for ATP synthesis without net production of NADPH. Therefore, one of these reactions must be deleted to ensure that the cycle is not functional.

Generally, we can conclude that the cMCSs of the GN and of the RN model follow the same fundamental design strategies to achieve coupled product and biomass synthesis. They block all potential AEF pathways or cycles that can potentially increase the ATP/NADPH balance above that of the LEF. Analysis of the GN model revealed that many more such potential pathways (beyond the “classical” AEF pathways) may exist at genome scale. The high number of 8 or even 14 reaction cuts computed for the RN and GN model, respectively, may appear unrealistic for strain construction and several reaction knockouts cannot or not easily be mapped to corresponding gene knockouts, such as photorespiration. However, some of the involved pathways to be blocked might already have a low capacity. For example, it has been reported that cyclic photosynthesis is highly active only under low-light conditions and the Mehler reaction is believed to play only a minor role in *Synechocystis* sp. PCC 6803 [54]. We therefore argue that already a subset of knockouts proposed by an cMCS might have beneficial effects for ethanol production, even though high growth-coupled ethanol yield cannot be guaranteed. Therefore, our findings help to identify potential bottlenecks and hidden bypasses that are relevant in conjunction with the optimization of cyanobacterial strains.

### Minimizing the number of required interventions: a role for ATP wasting mechanisms

We now seek to identify suitable intervention strategies that potentially reduce the large number of knockouts suggested by cMCSs analysis. As one of such, we may lower the threshold for the minimum guaranteed ethanol yield, e.g. from 0.03 mmol/mmol photons used in Figure 3B down to 0.02 mmol/mmol photons. This leads to a lowered number of target modes and increased number of desired modes which can then give rise to smaller cut sets [43]. Furthermore, implementation of overexpression candidates suggested by the CASOP analysis may also help to reduce the number of required cuts and to shift metabolic fluxes to desired behaviors. Since ATP wasting mechanisms were proposed as useful overexpression candidates by CASOP, we studied the effect of elevating the flux through the ATP maintenance reaction R35 (in the following abbreviated by ATPm). By this we mimic an increased ATP turnover enforced, for example, by futile cycles or other ATP consuming (wasting) mechanisms. We sampled the lower boundary of the ATPm flux in 20 equidistant steps from 0 to 14 mmol/gDW/h. We also explored the effect of moving the threshold for the minimum requested product yield by sampling this threshold between 0.002 and 0.04 mmol/mmol photons, again at 20 equidistant points, where the requested minimum biomass yield of 0.0001 gDW/mmol photons remained unchanged. We thus studied 20×20=400 different scenarios and calculated for each of them the resulting minimum size of the cMCSs, that is, the minimum number of reaction knockouts required to enforce coupled biomass and ethanol synthesis in the RN model. In all these calculations we set the upper boundary for the photon absorption rate to 100 mmol photons/gDW/h [40].

**Figure 6:**
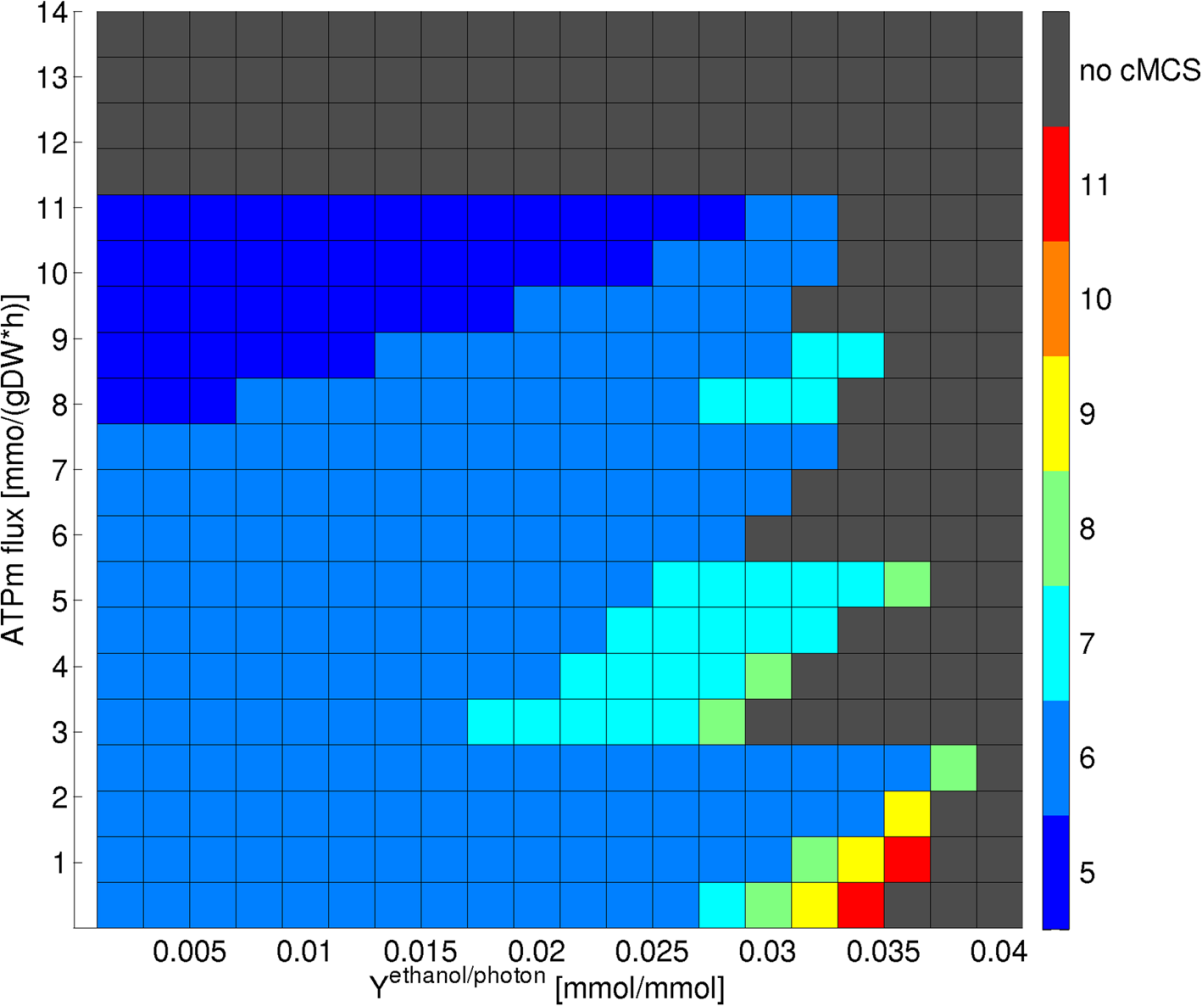
**Influence of the ATP maintenance demand (ATPm) and the requested minimal ethanol yield on the minimum number of required interventions in the resulting cMCSs.**

Figure 6 shows the results of this analysis. As expected, lowering the desired minimal ethanol yield for a fixed ATP maintenance demand reduces the number of required cuts, especially when moving from very high (0.03-0.04 mmol/mmol photons) to high (0.02-0.03 mmol/mmol photons) ethanol yield values. For example, in the case of a zero ATPm flux with minimum ethanol yield of 0.03 mmol/mmol photons (this scenario was used in previous section), at least 8 reactions must be disabled. This number can be reduced to 6 when demanding a slightly smaller ethanol yield of 0.026 mmol/mmol photons. In the other direction, if we demand a high product yield of at least 0.034 mmol/mmol photons, 11 reaction knockouts are necessary. The effects of increasing the ATPm flux for a fixed ethanol yield are less strict and in a few cases non-monotone. At moderate ATPm levels (0-2.5 mmol/gDW/h), we see that increasing this flux lowers the number of required interventions, e.g. from 8 to 6 for a minimum ethanol yield of 0.03 mmol/mmol photons. The maximal theoretical yield of growth-coupled ethanol synthesis is close to 0.038 mmol/mmol photons and only achievable with 8 reaction removals and a non-zero ATPm flux of 2.1 mmol/gDW/h. However, when increasing the ATPm flux along the requested ethanol yield of 0.028 mmol/mmol photons, we see that it first reduces the minimum number of knockouts from 7 to 6 before jumping up to 8 when the ATPm flux is 2.8 mmol/gDW/h. Increasing ATPm flux further decreases the minimal cMCS size down to 6 before increasing again to 7 when ATPm is at 8 mmol/gDW/h. These discontinuities arise because some cMCSs become invalid for larger ATPm fluxes as they can then not satisfy the minimum biomass yield constraint anymore. Only larger cMCSs might then remain valid. For the same reasons mentioned above, however, further elevating the ATPm flux again reduces the size of these cMCSs.

The minimal number of required reaction knockouts for growth-coupled ethanol synthesis is 5 which requires to have an ATPm flux between 8 and 11 mmol/gDW/h. Figure 6 also reveals that the ATPm flux cannot become arbitrarily high: for the used maximum photon uptake rate of 100 mmol photons/gDW/h, it should not exceed 11 mmol/gDW/h since otherwise coupling between product and biomass synthesis becomes infeasible for all product yields. Above this critical value, the LEF pathway with its relatively low ATP/NADPH ratio cannot generate sufficient amounts of ATP required for (i) the set ATPm flux, (ii) for the requested minimum biomass synthesis, and (iii) for ethanol synthesis (ethanol needed to consume NADPH in excess). In this case, the operation of AEF pathways becomes mandatory. However, as soon as AEF pathways are functional arbitrarily high ATP/NADPH ratios can be generated, and a guaranteed coupling of biomass and ethanol synthesis is therefore lost. The critical ATPm flux value depends on the maximal photon uptake rate; increasing the photosynthesis rate leads to larger acceptable ATPm levels.

One may speculate whether an increased ATPm flux could improve the ethanol yield also in the wild type without implementing any knockouts. We know that the wild-type alone cannot guarantee growth-coupled ethanol production. It is realistic to assume, however, that (1) cyanobacteria have optimized the fluxes along the LEF and AEF pathways for optimal growth in a given environment and (2) that an increased ATP consumption could not, or not immediately, be counterbalanced by the cell by re-adjusting the AEF fluxes. To study implications of such a scenario, we simulated optimal growth of cyanobacteria for a maximal photon uptake rate of 100 mmol photons/gDW/h, such that the cells adopt an optimal ratio between LEF and AEF fluxes that facilitates the theoretically maximal growth rate of 0.185 h ^−1^. We note that different AEF pathways are available and hence multiple optimal patterns for LEF and AEF fluxes exist that produce the optimal ratio required for maximal growth. In the following, the choice of any of these optimal solutions does not affect the argumentation. To study the effect of enforced ATP consumption we (1) select a growth-optimal solution, (2) freeze the exhibited fluxes of the AEF pathways, (3) increase the ATPm flux from 0 to 5 mmol/gDW/h, and (4) re-optimize again for maximal growth. We then observe that the ethanol production rate rises from 0 to 2.4 mmol/gDW/h (and the yield from 0 to 0.024 mmol/mmol photons) accompanied by a growth rate reduction from 0.185 to 0.072 h ^−1^. Hence, ethanol production becomes again mandatory if the cell cannot optimally re-adjust the fluxes along its AEF pathways upon increased ATP consumption.

### Intervention strategies for overproduction of isobutanol

To check how far the engineering strategies found for ethanol can be translated to other products, we recalculated EMs, CASOP scores, and cMCSs in the RN model with isobutanol as product of interest. The ATP/NADPH ratio of isobutanol is 1.0 (12 ATP/12 NADPH) and thus smaller than for ethanol. We found 126484 EMs and the maximal yield of isobutanol amounts to 0.02083 mmol/mmol photons corresponding to a theoretical demand of 48 photons per molecule isobutanol. The CASOP scores and resulting top knockout and overexpression candidates are similar to those obtained for ethanol overproduction. We also found that the 124 cMCSs calculated for ethanol would in the same manner enforce coupled growth and isobutanol synthesis ensuring for all these cMCSs a minimal isobutanol yield above 0.014 mmol/mmol photons and a minimum biomass yield of at least 0.0001 gDW/mmol photons. Hence, the strain design strategies found for ethanol are relevant for other biofuels as well, the latter acting as obligate redox sinks to adjust the ATP/NADPH ratio for biomass synthesis when AEF pathways are blocked.

## Conclusion

In this work, we investigated promising strain design strategies for the production of biofuels with cyanobacteria based on a genome-scale stoichiometric network reconstruction of *Synechocystis* sp. PCC 6803 [39]. Specifically, we constructed a reduced network model (RN) to identify the basic principles and intervention strategies that allow for an improved synthesis of ethanol, and then showed their general validity also in the full genome-scale (GN) model. Following related studies for heterotrophic microorganisms of biotechnological interest, our aim was to identify and characterize interventions that enforce coupled biomass and product synthesis. As a key result, and contrary to some previous studies, we found that such a coupling can be achieved also for phototrophic growth using appropriate interventions. Importantly, our suggested intervention strategies still allow for maintenance metabolism in the absence of light and do not rely on the assumption of optimal growth. The unifying principle behind all identified strategies is to force the cyanobacterium to use its photosynthetic apparatus to produce ATP and NADPH in a ratio that is below the required ratio for biomass synthesis. Thereby we enforce that desired products, such as ethanol, are utilized as a sink for the excess of reduction equivalents. A suitable strategy for achieving this goal is to block all AEF pathways and other metabolic routes and cycles that increase the ATP/NADPH ratio. The LEF becomes then the only mechanism for ATP and NADPH production whose maximal ATP/NAPH ratio of 1.28 is, as required, far below the minimal ratio of 1.51 required for growth. Analysis of cMCSs showed that a relatively large number of reactions must be blocked to ensure exclusive use of the LEF, mainly because of the many and poorly characterized alternative AEF pathways that exist in cyanobacteria. The large number of required interventions, the inability to suppress certain reactions, such as photorespiration or the Mehler-like reaction, and the fact that AEFs play an important role in balancing redox and energy metabolism in photosynthetic bacteria might give a pessimistic view on the practical realization of the proposed intervention design. However, we emphasize that our analysis has implications that go beyond a general understanding of how metabolic (especially electron) fluxes need to be redirected to achieve growth-coupled product synthesis. In particular, we hypothesize that already even subsets of the proposed reaction deletions will push the cell towards increased biofuel production. For example, exposing the cell to certain environmental regimes, such as high-light, may help to reduce the activity of AEF pathways indirectly.

In addition to knockout targets, we also identified certain fluxes or mechanisms whose upregulation would enhance product synthesis. Both CASOP and cMCS analysis clearly indicated that installing ATP wasting mechanisms could, at least partially, act as a substitute for deletions of AEF pathways. The increased consumption of ATP helps to keep the ATP/NADPH ratio low – again favoring or even demanding synthesis of a biofuel acting as redox sink. However, such an ATP wasting flux needs to be carefully controlled to not exceed certain thresholds.

Exemplified with ethanol and isobutanol, the identified intervention strategies and key principles of redirecting metabolic fluxes in cyanobacteria should be relevant for a large variety of biofuels and other products. Although our results were determined by a model of one representative of cyanobacteria, we believe that the drawn conclusions hold in an analogous fashion for other species of this bacteria class as well. Our results also suggest that achieving obligatory coupling of growth and biofuel (or other product) synthesis in photosynthetic bacteria requires different intervention strategies compared to heterotrophic organisms. In heterotrophic micro-organisms such as *Escherichia coli* or *Saccharomyces cervisiae* coupled biomass and product synthesis is usually achieved by growing the cells on a carbon substrate under anaerobic conditions and by deleting alternative fermentation pathways and fermentation products such that the desired product must be synthesized to balance reduction equivalents. In cyanobacteria, photosynthesis is used to generate ATP and NADPH needed to (i) fix carbon dioxide yielding precursors, and (ii) to produce biomass from ATP, NADPH and precursors. By introducing a disbalance between ATP and NADPH, some of the fixed carbon must be used to readjust the ATP/NADPH balance implying formation of ethanol or other (biofuel) byproducts.

## Additional files

Additional file 1
**Mapping of reaction IDs used in the main text to reaction IDs used in the RN and GN model.**

Additional file 2
**SBML code of the reduced network model (RN).**

Additional file 3
**SBML code of the genome scale model (GN).**

## Abbreviations

AEF: Alternate electron flow
ATPm: ATP maintenance demand
BM: Biomass
CASOP: Computational Approach for Strain Optimization aiming at high Productivity
MCS(s): Minimal cuts set(s)
cMCS(s): Constrained minimal cuts set(s)
EM(s): Elementary mode(s)
FNR: Ferredoxin NADP reductase
FQR: Ferredoxin plastoquinone reductase
gDW: Gram dry weight
GN: Genome-scale network model
LEF: Linear electron flow
MCS(s): Minimal Cut Set(s)
NDH: NAD(P)H dehydrogenase
PQ: Plastoquinone
PQH2: Plastoquinol
PSI: Photosystem I
PSII: Photosystem II
Cyd: Cytochrome *bd*-type quinol oxidase
Cox: *aa*3-type cytochrome *c* oxidase
RN: Reduced network model
SBML: Systems Biology Markup Language
