# Supplementary material for: Cyanobacterial biofuels: new insights and strain design strategies revealed by computational modeling
Source: Microb Cell Fact. 2014 Sep 19;13:128. doi: 10.1186/s12934-014-0128-x (PMC4180434; doi:10.1186/s12934-014-0128-x)
Supplement: Additional file 1 — Mapping of reaction IDs used in the main text to reaction IDs used in the RN and GN model. [file 12934_2014_128_MOESM1_ESM.pdf]

Additional file 1

Cyanobacterial biofuels: new insights and engineering strategies revealed by computational modeling

Philipp Erdrich[1], Henning Knoop[2], Ralf Steuer[2], Steffen Klamt[1*]

1 Max Planck Institute for Dynamics of Complex Technical Systems, Magdeburg, Germany
2 Humboldt-University of Berlin, Institute for Theoretical Biology, Invalidenstrasse 43, 10115 Berlin, Germany
* Email: klamt@mpi-magdeburg.mpg.de

Table S1: Mapping of reaction IDs used in the main text to reaction IDs used in the RN and GN model files.

Reaction ID	Reaction ID (Model)	Reaction ID	Reaction ID (Model)
R1	PR0004	R20	GM0016
R2	PR0006	R21	GM0017
R3	PR0014	R22	GM0025
R4	PR0018	R23	R20131
R5	Mehler	R24	R20132
R6	GE0010	R25	R20133
R7	FQR	R26	PT0011
R8	PP0016	R27	PP0020
R9	TR0049	R28	PP0021
R10	PT0027	R29	EtOH-ex
R11	TR0053	R30	PT0009
R12	GE0009	R31	PDC
R13	PT0026	R32	PT0001
R14	GS0043	R33	TR0003
R15	GS0042	R34	GS0006
R16	GS0041	R35	GE0001
R17	GS0040	R36	ST0016
R18	GM0010	R37	ST0014
R19	GM0011	R38	PP0024
